# Experimental Assessment of the Effect of Temperature in the Range of 20–80 °C on Structural Behaviour of NSM CFRP Reinforced Concrete Slabs

**DOI:** 10.3390/ma19071382

**Published:** 2026-03-31

**Authors:** Patrícia Silva, Hevar Hamid Abdulrahman, Gonçalo Escusa, Luís Correia, Miguel Azenha, José Sena-Cruz

**Affiliations:** Institute for Sustainability and Innovation in Structural Engineering (ISISE), Advanced Production and Intelligent Systems Associated Laboratory (ARISE), Department of Civil Engineering, University of Minho, 4800-058 Guimarães, Portugal; patricia.msilva@outlook.pt (P.S.); hevarrahman@yahoo.com (H.H.A.); g.escusa@gmail.com (G.E.); lcorreia@civil.uminho.pt (L.C.); miguel.azenha@civil.uminho.pt (M.A.)

**Keywords:** carbon fibre-reinforced polymer (CFRP), near-surface mounted (NSM), elevated temperature, reinforced concrete slabs

## Abstract

The near-surface mounted (NSM) technique with carbon fibre-reinforced polymer (CFRP) composites has been proven to be one of the most effective alternatives for the flexural strengthening of existing reinforced concrete (RC) members. However, several issues remain unresolved, including the effects of elevated temperatures on the performance of these strengthened RC elements. This study experimentally investigates the mechanical performance of RC slabs strengthened with NSM-CFRP systems under elevated temperatures, using both (i) steady-state and (ii) transient heating under applied loads. The steady-state tests were conducted at 20, 40, 50, 70, and 80 °C, while the transient tests were performed at 20 and 80 °C. Deflections, strains, temperatures and loads were registered during the heating phase and during the flexural tests up to failure. These measurements were used to analyse the system response in terms of load–deflection curves, evolution of concrete and CFRP strains, and bond stresses between the epoxy adhesive and CFRP. At 80 °C, the NSM-CFRP-strengthened RC slabs exhibited an average reduction of 12.1% (steady-state) and 2.3% (transient) in ultimate strength. Moreover, the concrete crushing failure mode governed up to 70 °C, despite passing the epoxy’s glass transition temperature (54 °C), while cohesive failure of the adhesive governed the failure at 80 °C.

## 1. Introduction

Carbon fibre-reinforced polymer (CFRP) composites have been increasingly used in various fields of engineering in the past few decades [1]. One of their most prominent applications is the strengthening of reinforced concrete (RC) structures [2]. CFRP composites have several advantages over traditional strengthening materials, such as lightness, ease of installation, high strength-to-weight ratio and resistance to corrosion [3]. Among the CFRP composites’ strengthening techniques, the near-surface mounted (NSM) and externally bonded reinforcement (EBR) techniques are the most widely adopted [1,3]. In NSM, FRP laminates or rods are inserted inside grooves cut through the concrete cover of the RC member and bonded with an adhesive, typically epoxy-based. In EBR, FRP composites are adhesively bonded to the exterior surface of the RC member, which has been previously prepared. The NSM technique provides more efficient stress transfer for a given reinforcement geometry as the reinforcement is bonded on all faces rather than one, reducing premature debonding and allowing better utilisation of the reinforcement compared to EBR (with or without prestressing) [3,4,5,6,7,8]. The NSM technique also requires less preparation work and offers better protection of the FRP against external aggressive agents, environmental conditions, and vandalism [5,7].

A critical factor in structural strengthening systems is the exposure to high service temperatures and fire, particularly for cold-curing epoxy adhesives due to their low glass transition temperature (*T_g_*) [3]. When the epoxy approaches a temperature equal to or higher than the *T_g_*, a sudden change in its mechanical properties occurs from a glassy, hard and relatively brittle state into a rubber-like state. This transition from solid to a viscous state is a continuous process over a certain temperature range (starting at about 10–20 °C below the *T_g_*), degrading bond performance [9,10]. Certain structures, such as bridges, are susceptible to high ambient temperatures (close to 80 °C) due to the sealing layer and asphalt application [11].

Research on the performance of strengthened RC systems at elevated temperatures can generally be classified into two main areas: (i) fire resistance testing (up to ~1000 °C in a fire furnace) evaluating structural integrity under extreme thermal conditions and (ii) high service temperature testing (<100 °C) evaluating loading capacities at a target temperature near or above the *T_g_* of the adhesive.

Studies on the fire resistance of various slab-strengthening techniques showed that NSM-CFRP systems have a higher average critical temperature when the CFRP debonded (2.9 to 4.9 times *T_g_*) compared to the EBR (1.1 to 1.6 times *T_g_*), likely due to the better confinement within the concrete cover [1,12]. However, tests conducted showed the temperature susceptibility of both techniques without insulation, losing their effectiveness after exposure for 2 min for EBR and 16 min for NSM, while insulation systems increased the endurance time up to 30 min for EBR and 90 min for NSM reinforcement [12]; that is why insulation systems and techniques have been investigated on multiple occasions, e.g., [3,12,13]. Fire endurance tests have been developed using the NSM, focusing on the type of adhesive used for bonding with RC beams, comparing epoxy and cement-based adhesives, with epoxy showing lower performance [13,14,15]. In Ref. [15], the CFRP composite laminates started to gradually lose their contribution at around 300 °C using epoxy adhesives, compared to 830 °C for cement-based ones.

High service temperature research includes sustaining service loads [16], comparing the behaviour of epoxy and cement-based adhesives [16], single- and double-lap shear tests on the adhesives [17,18], and the flexural performance of strengthened beams [1,19]. In both the flexural and adhesive shear tests using NSM, the failure mode changes from cohesive in the RC member to adhesive in the interface between the RC and CFRP near *T_g_* [1,17,18,19]. Additionally, cement-based adhesives outperform epoxy adhesives under elevated service temperatures ranging from 50 to 255 °C [16,17,18]. Under similar conditions, NSM shows superior performance over EBR, consistent with observations from the fire endurance tests [20].

Experimental programmes comparing the NSM flexural performance under elevated service temperature include tests at: (i) ambient temperature and 40 °C—well below the adhesive’s *T_g_* (53.9–65.3 °C)—and (ii) elevated temperatures of 60, 70, and 85 °C [1,19]. Performance is only marginally affected up to a temperature of 60 °C, but ultimate capacity drops by 3.95% at 70 °C and 10.45% at 85 °C [1]. Additionally, failure evolves from FRP rupture at 20, 40, and 60 °C, to end debonding at 70 °C and concrete crushing at 85 °C. This change in failure behaviour can be attributed to the degradation of the mechanical properties of both the concrete and the epoxy at elevated temperatures [1].

Most studies to date focus on strengthened beams, leaving the flexural performance of NSM-strengthened slabs under elevated service temperatures largely unexplored. While experiments on RC beams are well-documented, only a few studies have investigated slab strips subjected to fire [3,12]. This gap motivates the present investigation of NSM CFRP-strengthened RC slabs in the range of 20–80 °C.

The main goal of the present study is to contribute to the current state of knowledge on the effect of elevated service temperatures on the NSM technique. This is done through an experimental investigation of RC slab strips strengthened with NSM CFRP laminates submitted to elevated temperatures in the range of 20–80 °C, spanning above and below the epoxy adhesive’s *T_g_*. The experimental programme consisted of nine slabs tested under two conditions: (i) steady-state and (ii) transient. In the steady-state tests, slabs were first heated to a predefined temperature (20, 40, 50, 70, and 80 °C) and then monotonically four-point bending loaded to failure. For the transient tests, slabs were initially preloaded to 2/3 of their ultimate load at room temperature, followed by a gradual increase in temperature up to 80 °C, unloaded, and monotonically tested to failure. The following section presents a detailed description of the experimental programme.

## 2. Specimens, Materials and Methods

### 2.1. Tests at Elevated Temperature with Slabs

#### 2.1.1. Setup and Instrumentation

**Figure 1:**
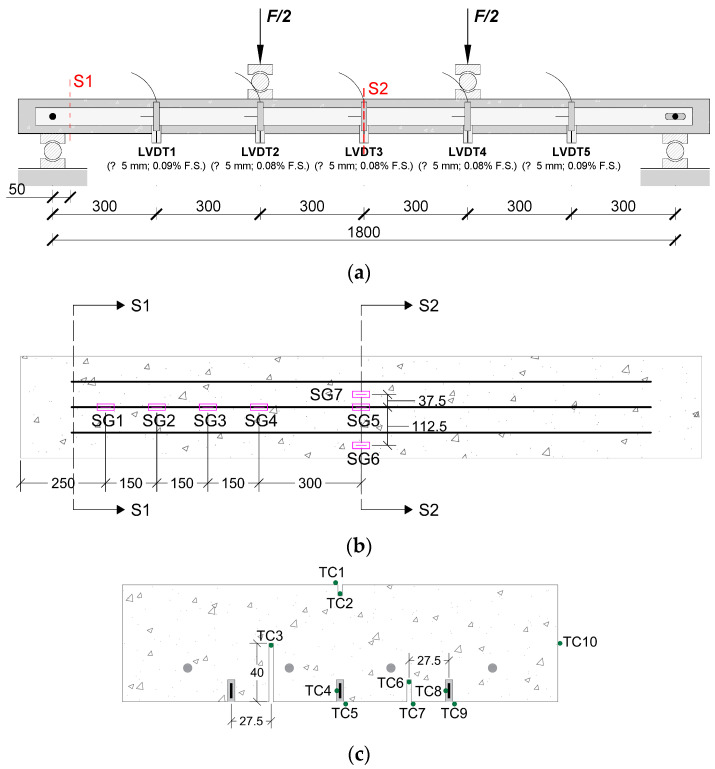
Experimental setup and instrumentation scheme: (**a**) four-point bending test arrangement with LVDTs; (**b**) strain gauge (SG—pink boxes) positions with cross-sections shown; (**c**) cross-sectional view of S1 and S2 showing thermocouple (TC—green dots) distribution. Note: all the dimensions are in [mm].

The experimental setup and instrumentation scheme are depicted in Figure 1. A four-point bending setup with a shear span of 600 mm and a total span of 1800 mm was used, as shown in Figure 1a. Deflections were recorded using five Linear Variable Displacement Transducers (LVDTs, RDP Electronics Ltd., Wolverhampton, UK) positioned at the mid-span, load application points, and midpoints between supports and loads. Seven strain gauges (TML BFLA-5-3-3L, Tokyo Measuring Instruments Laboratory Co., Ltd., Tokyo, Japan) were installed on the CFRP laminates (SG1–SG5) and steel bars (SG6–SG7), while one additional gauge (TML PFL-30-11-3L, Tokyo Measuring Instruments Laboratory Co., Ltd., Tokyo, Japan) monitored concrete strains, as shown in Figure 1b. The applied load (*F*) was measured by a 200 kN load cell positioned between the actuator and the grip, featuring a linearity error below ±0.05% F.S.

K-type thermocouples were placed at two instrumented sections: (i) a section 50 mm from the support (S1) and (ii) the mid-span (S2), as shown in Figure 1b. A total of 25 thermocouples were installed to monitor temperatures at various points, as illustrated in Figure 1c: 10 thermocouples per section (S1 and S2) and 5 to measure the air temperature inside and outside the climatic chamber. Temperature data were recorded using DataTaker DT85M [21] and PicoLog USB TC-08 [22] systems.

**Figure 2:**
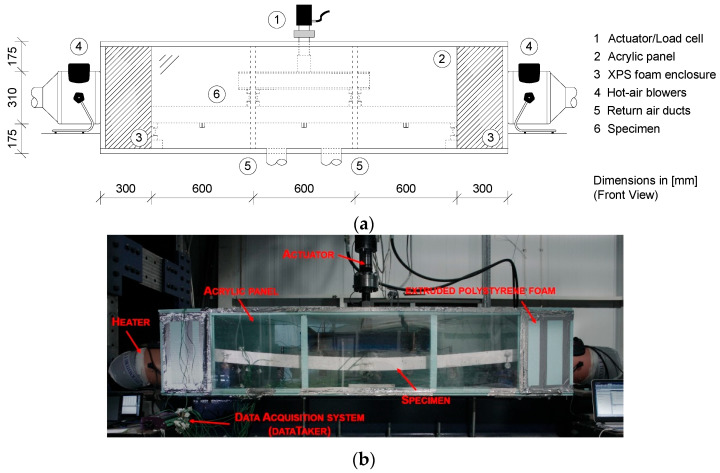
(**a**) Schematic diagram of the climatic chamber and (**b**) the setup used.

Figure 2 shows a general view of the fabricated chamber and heating system used for the steady-state (SS) and transient (TR) tests. The chamber was constructed from extruded polystyrene foam and designed to withstand temperatures of up to 100 °C. Two industrial hot-air blowers were used to heat the interior, with temperature controlled by an Arduino UNO [23] equipped with a MAX31855 thermocouple amplifier ADC featuring cold-junction compensation [24]. After describing the instrumentation and chamber setup, the experimental programme is detailed below.

#### 2.1.2. Test Programme

**Figure 3:**
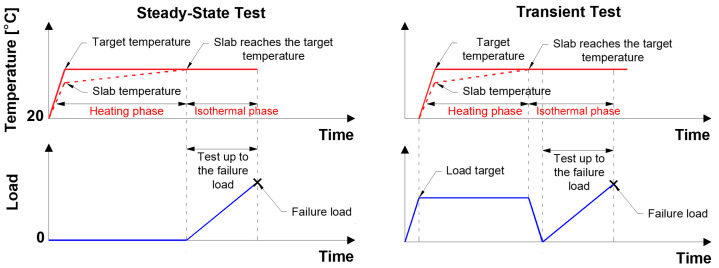
Temperature and load evolution over time throughout the steady-state and transient tests [25].

As previously referenced, the present experimental programme encompasses steady-state (SS) and transient (TR) tests. Figure 3 illustrates schematically the temperature and loading protocols adopted, while Table 1 provides further details, in addition to the denomination that was adopted in each test.

Six SS slabs were tested at 20, 40, 50, 70, and 80 °C, with two slabs at 80 °C for repeatability. The SS slabs were heated without a sustained load until the target temperature. Subsequently, the slabs were monotonically loaded to failure with a rate of 0.02 mm/s while maintaining the target temperature.

Two slabs were used in the TR tests: (i) one of the TR slabs (SL1_TR-80) was exposed to approximately 80 °C for 4 h, while (ii) the other slab (SL2_TR-80) was exposed for nearly 12 h, corresponding to the time required for the slab to reach 80 °C. Prior to the initiation of the heating phase, the TR slabs were preloaded to 2/3 of their ultimate capacity, with a rate of 14 N/s, at room temperature (20 °C). After the heating phase, the TR slabs were unloaded at 0.1 mm/s and then loaded to failure under the same displacement control as the SS tests.

Additionally, a control specimen (SL1_TR-20) was tested monotonically to failure at room temperature (20 °C).

### 2.2. Specimen Design

**Figure 4:**
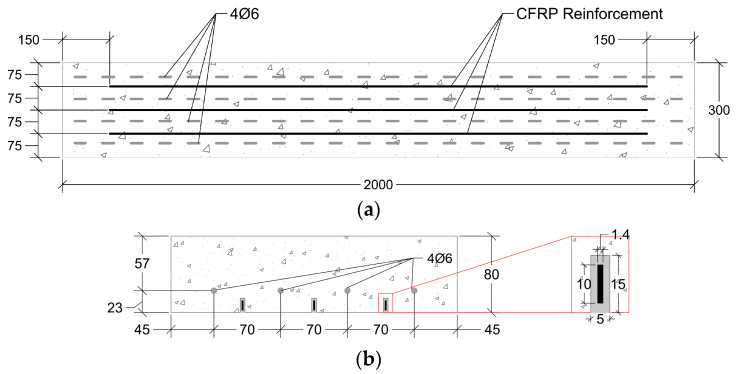
Slab geometry and reinforcement details: (**a**) bottom view, (**b**) longitudinal cross-section. Note: all the dimensions are in [mm].

The experimental programme consisted of nine slabs with dimensions of 2000 mm in length, 300 mm in width, and 80 mm in thickness, as shown in Figure 4. The longitudinal reinforcement comprised four 6 mm diameter rebars, corresponding to a longitudinal reinforcement ratio of 0.47%, as illustrated in Figure 4a. The NSM technique was adopted for flexural strengthening, using three CFRP laminate strips, which provided an equivalent longitudinal reinforcement ratio of 0.68% in total [26]. Details of the groove geometry and corresponding reinforcement configuration are presented in Figure 4b.

The strengthening of the slabs was carried out approximately three months after concrete casting. Before strengthening, grooves were cut using a saw-cutting machine—the average groove width and depth were 5.47 mm (CoV = 1.82%) and 15.59 mm (CoV = 2.16%), respectively. The strengthening procedure involved the following steps: (i) cleaning the grooves with compressed air and the CFRP laminates with acetone; (ii) preparing the epoxy adhesive according to the manufacturer’s recommendations; (iii) applying the adhesive in the grooves and on the lateral faces of the laminates using a hand scraper; (iv) carefully inserting each laminate at the centre of the groove; and (v) smoothing the external surface of the groove region. The strengthening process was performed under laboratory conditions, with an average temperature of 25 °C and a relative humidity of 42%. Further details relating to these slabs, including the rationale supporting the geometry, materials, reinforcements and strengthening solutions, can be found in the following references [27,28,29]. The RC slab geometry, longitudinal reinforcement, groove dimensions, material properties, and strengthening procedure adopted in the present study are identical to those used in a companion publication examining the fatigue performance of the same NSM-CFRP-strengthened slabs [28]. Both investigations form part of the same R&D project. Further details can be found in [27].

### 2.3. Materials

#### 2.3.1. Concrete

Only one batch was used to cast all the specimens involved in this experimental programme. The concrete mixture was composed of 765.3 kg/m^3^ of coarse aggregate (maximum aggregate size of 12.5 mm), 125.8 kg/m^3^ of fine aggregate, 345.8 kg/m^3^ of fine sand, 562.3 kg/m^3^ of coarse sand, 212.5 kg/m^3^ of cement 42.5 type II, 143 kg/m^3^ of fly ash, 124 L/m^3^ of water, and 2.9 kg/m^3^ of plasticiser (Chryso^®^Plast 820, Chryso Portugal, Baguim do Monte, Portugal).

The mechanical characterisation of the concrete was carried out using five cylindrical specimens with a diameter of 150 mm and a height of 300 mm. The specimens were tested in compression at the same time as the slab tests. The modulus of elasticity and compressive strength were evaluated in accordance with LNEC E397-1993:1993 [30] and NP EN 12390-3:2011 [31], respectively. The average compressive strength of the concrete was 51.9 MPa (CoV = 3.9%), and the corresponding Young’s modulus was 28.8 GPa (CoV = 1.5%).

#### 2.3.2. Steel Reinforcement

The longitudinal reinforcement was grade A500 NR, in accordance with NP EN 1992-1-1 [32]. Tensile tests were conducted following NP EN 10002-1:1990 [33] to determine the mechanical properties of the steel. The average values of Young’s modulus, hardening modulus, and ultimate strength were 212.2 GPa (CoV = 6.3%), 0.7 GPa (CoV = 6.6%), and 733.0 MPa (CoV = 1.0%), respectively.

#### 2.3.3. CFRP Laminate

The CFRP laminate strips were supplied by S&P Clever Reinforcement Company under the trademark CFK 150/2000 (S&P Clever Reinforcement Company AG, Seewen, Switzerland). They consisted of unidirectional carbon fibres bound with an epoxy vinyl ester resin. Each laminate had a rectangular cross-section of 10 mm × 1.4 mm and a smooth external surface. The mechanical properties were evaluated in accordance with ISO 527-5:1997 [34]. The measured average values of Young’s modulus, tensile strength, and strain at peak stress were 169.5 GPa (CoV = 2.5%), 2648.3 MPa (CoV = 1.8%), and 1.6% (CoV = 1.8%), respectively.

#### 2.3.4. Epoxy Adhesive

The epoxy adhesive used to bond the CFRP laminates to the RC slabs was S&P Resin 220. It is a solvent-free, thixotropic, grey, two-component system (resin and hardener), recommended for application between 10 °C and 35 °C. After mixing, the compound density ranges between 1.70 and 1.80 g/cm^3^. Following three days of curing at 20 °C, the manufacturer reports: (i) compressive strength > 70 MPa; (ii) tensile E-modulus > 7.1 GPa; (iii) shear strength > 26 MPa; and (iv) adhesive tensile strength to concrete or CFRP > 3 MPa [35]. Previous studies developed by the authors indicate that the adhesive attains about 90% of its maximum stiffness after 18 h of curing at 20 °C, a tensile strength of 21.98 MPa (CoV = 0.87%), and a tensile E-modulus of 7.10 GPa (CoV = 5.74%) [36,37].

**Figure 5:**
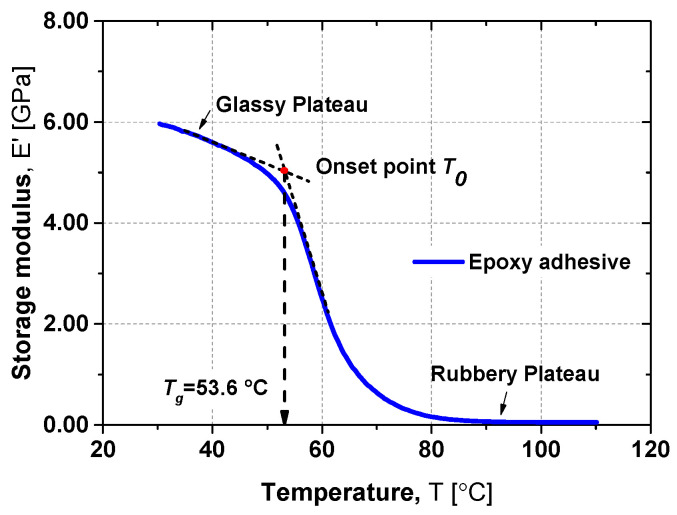
*T_g_* of epoxy adhesive based on the onset of the drop in the storage modulus curve.

The *T_g_* was determined following the procedure in [10] (more details in [9,38]), based on the onset of the drop in the storage modulus curve, Figure 5. The obtained *T_g_* was 53.6 °C, with a marked reduction in storage modulus above this temperature, reflecting the transition to viscoelastic behaviour of the polymer matrix.

## 3. Results and Discussion

### 3.1. Steady-State Tests

**Figure 6:**
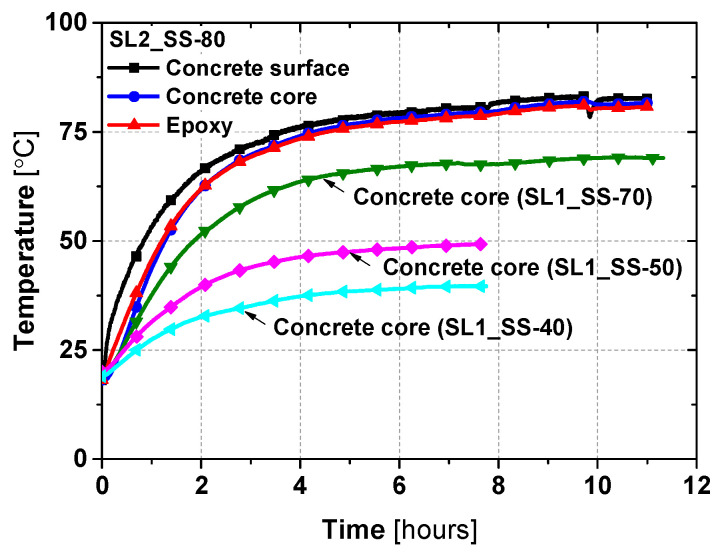
Typical temperature evolution curves for different slabs.

The typical evolution of the air temperature and the average temperatures measured at the two instrumented cross-sections (S1 and S2) for slab SL2_SS-80 are shown in Figure 6. The surface temperature rises earlier than that of the core and epoxy, mainly due to differences in thermal conductivity, before a homogeneous target temperature is eventually reached throughout the section. For the remaining slabs (SL2_SS-40, SL2_SS-50, SL2_SS-70), a similar temperature correlation was observed; therefore, only the concrete core temperature evolution is presented. The additional thermocouples (TC1, TC2, and TC5–TC10) installed at the two cross-sections were used solely for verification purposes, and their recorded temperatures were nearly identical to those shown. This agreement confirms the thermal homogeneity across the concrete section.

The heating process comprised an initial linear rise lasting about one hour, reaching approximately 60% of the target, followed by an asymptotic phase. Specifically, slabs SL1_SS-80, SL2_SS-80, and SL1_SS-70 reached thermal equilibrium in about 10 h, i.e., all thermocouples reached the target temperature, whereas SL1_SS-40 and SL1_SS-50 required only about 6.5 h. Despite different target temperatures, all slabs exhibited a similar temperature evolution, consistent with the existing literature, e.g., [39].

**Figure 7:**
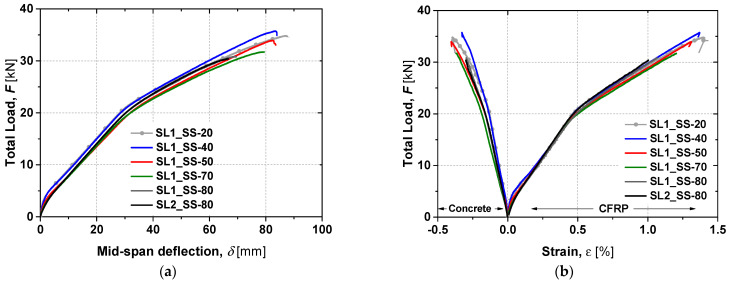
(**a**) Total load versus mid-span deflection curves and (**b**) total load versus strain curves of flexural tests up to failure for the SS slabs [27].

**Figure 8:**
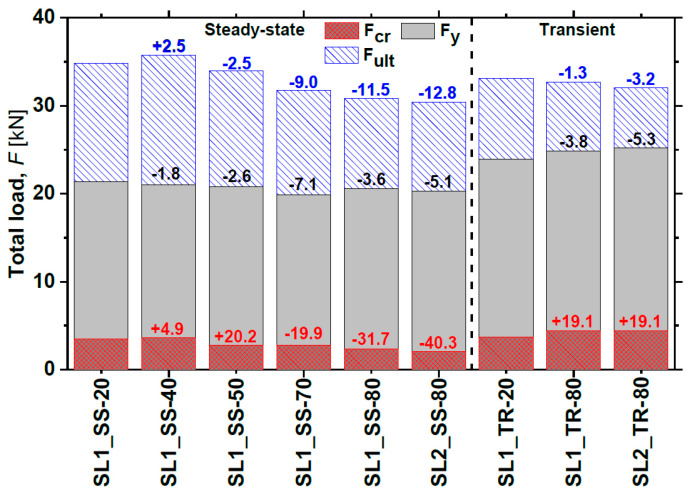
Notable points of all tested slabs and variation of ultimate capacity with respect to the corresponding reference slabs (SL1_SS-20 and SL1_TR-20) [25].

The total load–deflection curves (*F*-*δ*) curves and the strain evolution in the CFRP and in the concrete up to failure are presented in Figure 7. Key load–deflection parameters are summarised in Table 2, while Figure 8 illustrates the values of *F_cr_*, *F_y_* and *F_max_* and their variations relative to the control slab SL1_SS-20.

**Figure 9:**
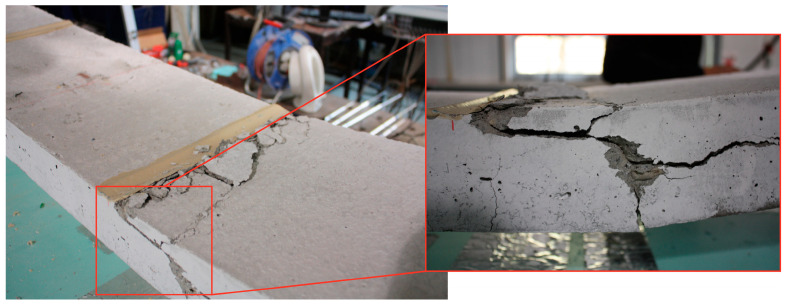
Representative concrete crushing failure mode observed in slabs tested between 20 °C and 70 °C (SL1_SS-50 shown).

**Figure 10:**
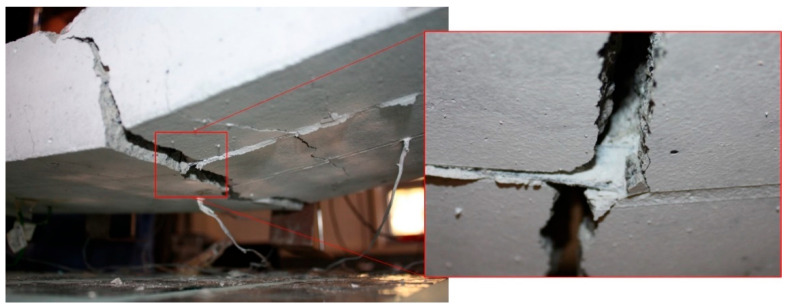
Cohesive failure observed at the adhesive at 80 °C (SL1_SS-80 shown).

The *F*-*δ* curves show the typical three-stage response of strengthened slabs: (i) uncracked concrete, (ii) cracked concrete, and (iii) steel yielding. Slab SL1_SS-40 achieved the highest ultimate load and stiffness, even exceeding the reference. Slab SL1_SS-50 also showed higher stiffness in the third branch despite a lower ultimate capacity, whereas slabs at 70 °C and 80 °C exhibited reduced stiffness in both the first and third branches. The maximum concrete strain reached was approximately 0.4% in slabs SL1_SS-20 and SL1_SS-50, while a maximum CFRP strain of about 1.4% was recorded in SL1_SS-20 and SL1_SS-40. Slabs tested between 20 °C and 70 °C failed by concrete crushing, with typical mid-span flexural cracks propagating symmetrically until sudden top-face crushing (Figure 9). Although the epoxy adhesive had a *T_g_* of around 54 °C, the overall structural response remained largely unaffected up to 70 °C. No visible debonding of the CFRP laminates or adhesive failure was observed, confirming effective stress transfer. However, cohesive failure of the epoxy occurred at 80 °C (see Figure 10), leading to a 21% reduction in CFRP strain at ultimate load and a corresponding 12% drop in load-carrying capacity. Furthermore, the ultimate load-carrying capacity decreased by 9, 12 and 13% for slabs SL1_SS-70, SL1_SS-80 and SL2_SS-80, respectively, when compared with the reference slab.

### 3.2. Transient Tests

**Figure 11:**
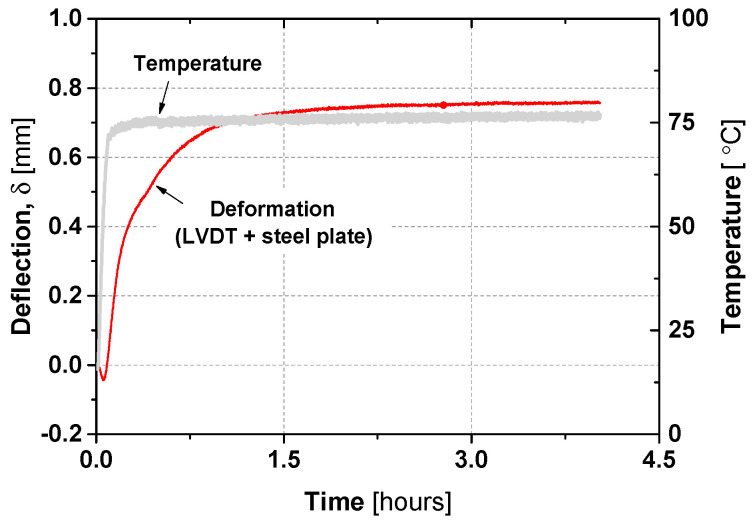
Time versus deflection of the LVDT due to the effect of temperature in the LVDT itself and the steel plate placed to measure the displacements in the slab test [27].

Although the LVDTs are rated for operation between −50 and 120 °C, unexpected behaviour was observed during the transient slab tests—an almost instantaneous increase in measured deformation when the heaters were activated. To quantify this effect, an experimental artefact with the LVDT and a steel plate equal to the ones used in the test of the slabs was exposed to 80 °C for ~4 h under similar temperature conditions to the slab tests. Figure 11 shows the time evolution of the measured deformation: an initial contraction occurred as the air temperature increased, followed by an expansion over roughly 1.5 h, mainly due to steel plate heating and sensor response. The maximum deviation recorded due to temperature effects from 20 °C to 80 °C was 0.75 mm. Given that this value is ~1% of the deflections recorded at maximum load, no thermal compensation was applied to the readings, as the uncertainty remains negligible relative to the structural response.

**Figure 12:**
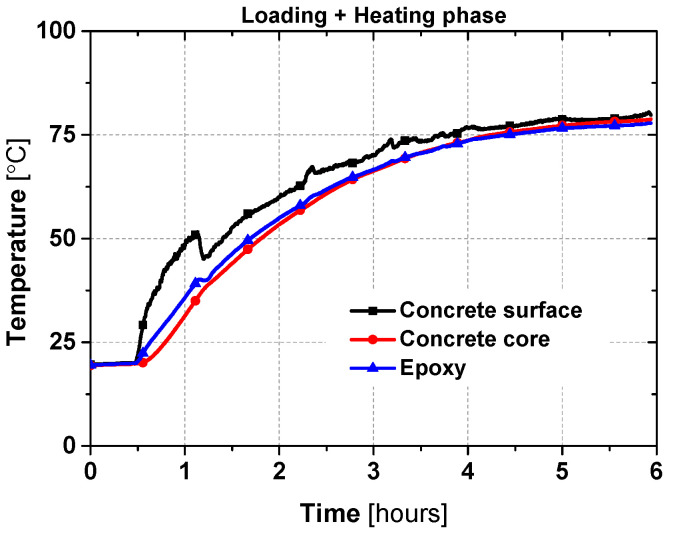
Time versus temperature of slab SL1_TR-80 [27].

Figure 12 depicts the temperature evolution of the epoxy, concrete surface, and slab core for SL1_TR-80 during the heating phase. Some instability in the air temperature occurred due to a heater issue (between 1 h and 1.5 h), but the test was not compromised, given the visually observed responses—the temperature evolution in the materials followed the same trend as observed in the steady-state tests.

**Figure 13:**
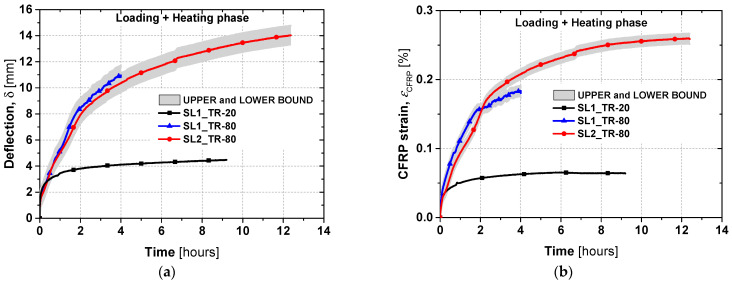
Time versus (**a**) deflection and (**b**) CFRP strain during the heating phase.

Figure 13 shows the evolution of deformations and CFRP strains under sustained load during the heating phase. Uncertainty arose in the measured deformations due to the temperature effects on the LVDTs and the steel plate device. To address this, upper and lower bounds were calculated by adding and subtracting 0.75 mm—the maximum temperature-induced LVDT deviation—to the measured values, defining the range of possible deformations. The same approach was applied to the CFRP strain measurements and temperature-compensation data from the strain gauge technical sheet at 80 °C. Figure 13b plots the CFRP strain evolution caused by the combined effects of sustained load (creep) and temperature variation in the slab. The gradual strain increase over time reflects both the creep of the concrete and the thermal expansion of the composite.

For all three tested slabs, the deformation increased over time. However, the difference between the slabs tested at 20 °C and 80 °C is pronounced: the reference slab (SL1_TR-20) reached deformations of 4.1 mm and 4.3 mm after approximately 4 and 9 h, respectively, whereas the heated slabs (SL1_TR-80 and SL2_TR-80) exhibited much larger deformations of about 11.0 mm and 13.1 mm over the same time intervals.

**Figure 14:**
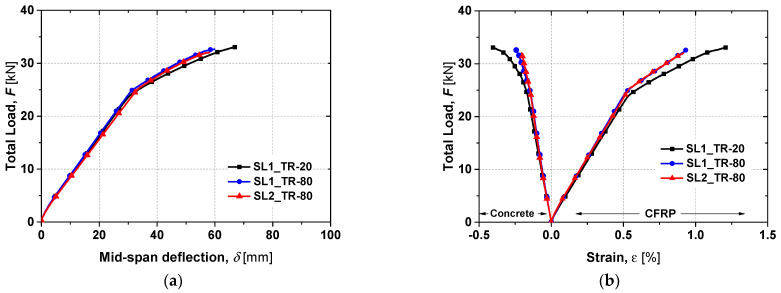
(**a**) Total load versus mid-span deflection and (**b**) total load versus strains for flexural tests up to the failure of TR slabs.

Figure 14 presents the static test results up to failure for the TR slabs. A linear response up to yielding reflects pre-existing cracking caused by the sustained load. In this phase, the slabs tested at 80 °C exhibited larger linear deformations (>8%) compared to the reference slab SL1_TR-20, while the stiffness of the first branch remained similar for both the heated and reference slabs.

The behaviour in the second branch was similar for both slabs tested at 80 °C, though marginally stiffer than the reference slab. The ultimate load of SL1_TR-80 and SL2_TR-80 decreased only by 1.3% and 3.2%, respectively, compared to SL1_TR-20. The slight difference between the two heated slabs can be attributed to the different durations under sustained load and elevated temperature. The strain evolution shown in Figure 14b indicates a maximum CFRP strain of 1.4% and a concrete strain of 0.4% for SL1_TR-20, which failed by concrete crushing. In contrast, SL1_TR-80 and SL2_TR-80 exhibited cohesive failure of the epoxy, similar to the SS slabs tested at 80 °C. Their maximum CFRP strains were 1.27% and 1.23%, respectively—approximately 12% lower than the reference slab. These results provide a basis for evaluating the influence of elevated temperature on structural performance, further discussed in the next section.

### 3.3. Discussion

Starting with the results of the SS tests, the superior performance of slab SL1_SS-40 can be attributed to post-curing of the epoxy adhesive during the heating phase. Post-curing typically occurs when epoxy adhesives are exposed to temperatures higher than those used during their initial cure, leading to enhanced mechanical properties due to increased chain branching and molecular crosslinking in any remaining unreacted groups [40]. This is consistent with experimental observations from pull-out tests on NSM-CFRP-strengthened concrete specimens cured at 20 and 40 °C, where curing at 40 °C significantly improved the bond strength performance of the studied NSM-CFRP system and also significantly improved the final elastic modulus of the epoxy due to enhanced crosslinking of the polymeric chains [41]. In addition, the concrete–epoxy and epoxy–CFRP adhesion mechanisms can be improved at temperatures approaching the *T_g_*. The increase in temperature may have strengthened the chemical–covalent bonding within the adhesive, either through the formation of new bonds or reinforcement of existing ones between the polymer matrix and the CFRP laminate [42].

Previous studies indicate that the temperature range investigated here only marginally affects concrete behaviour [29,43]. Thus, the stiffness reduction observed at 70 °C and 80 °C likely results from thermal expansion and consequent internal microcracking [43], combined with the degradation of epoxy properties above its *T_g_*, where stiffness decreases by about 93% (see Section 2). Despite this significant reduction in adhesive stiffness, the ultimate load capacity was only slightly affected. This is due to the stress transfer mechanism inherent to NSM CFRP systems, where tensile and compressive forces develop through interfacial shear stresses at the adhesive–concrete and CFRP–adhesive interfaces. These micro-mechanisms allow effective load transfer even when the adhesive has very low stiffness. For example, a tensile Young’s modulus as low as 8 MPa has been shown to provide satisfactory performance in similar systems [44,45].

**Figure 15:**
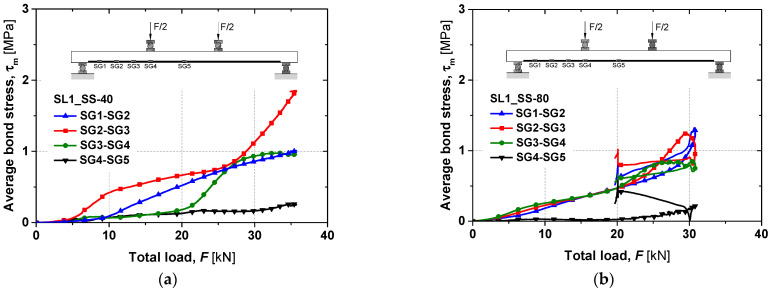
(**a**) Average bond stress versus total load curves of SL1_SS-40 and (**b**) SL1_SS-80 for flexural tests up to failure.

To complement the global load–deflection analysis, the bond behaviour during the monotonic static tests up to failure was further examined. The average bond stress between consecutive strain gauges was evaluated following the procedure described in [28]. This method assumes perfect bonding between materials and linear elastic behaviour of the CFRP. It should be noted that due to the adhesive’s highly non-linear (rubbery) behaviour at temperatures exceeding *T_g_*, it may violate these assumptions, and some uncertainties are introduced. Nevertheless, consistent with observations mentioned in the preceding paragraph on the persistence of stress transfer through interfacial shear mechanisms, it was assumed that the approach remains applicable for comparative evaluation across temperatures, providing approximate values of the average bond stresses. The resulting average bond stress–total load curves at different locations for slabs SL1_SS-40 and SL1_SS-80 are shown in Figure 15. In both slabs, the bond stresses in the pure bending region were minimal, as expected due to the constant bending moment (strain gauges SG4 and SG5). However, between the support and the load application point, the slabs exhibited distinct bond behaviour. For SL1_SS-80, the bond stresses between SG1–2, SG2–3, and SG3–4 show an almost linear distribution, in contrast to SL1_SS-40. The literature suggests that this response is associated with the epoxy adhesive exceeding its *T_g_*, which promotes a more uniform shear stress distribution along the bonded length [17]. The difference in bond stresses between the two slabs can be attributed to an increase in the effective bond length at elevated temperatures. In SL1_SS-80, the bond strength, which depends on transverse confinement and chemical adhesion, was further enhanced by friction at the CFRP–adhesive interface, as the adhesive had essentially completed its glass transition [17]. The observed failure mode for SL1_SS-80 was cohesive shear within the adhesive, governed primarily by its mechanical properties, particularly shear strength, and the degree of transverse confinement [5].

For the TR tests, the increase in measured deformation during the heating phase is primarily due to the expansion of the steel plate device, which has high thermal conductivity. The continued evolution of deformation for about 1 h suggests that electronic components within the LVDT sensor also influenced the readings. The LVDT operates in a 4-wire configuration, connected to a dedicated signal-conditioning circuit that converts its AC output into usable DC voltage. The wiring and signal-conditioning architecture affect both measurement simplicity and instrumentation cost. Temperature variations can further impact LVDT performance through mechanical expansion, shifts in winding resistance, and changes in core and coil material properties, potentially causing zero-shifts or scale-factor changes for a given displacement [46]. This observation further justifies the upper and lower deformation bounds developed in the previous section to account for temperature-induced artefacts.

The higher deformations observed in the slabs exposed to 80 °C can be attributed to the combined effects of concrete creep and the reduced stiffness of the epoxy adhesive. Quantifying the individual contributions of these two mechanisms is challenging, as creep deformation increases with temperature, while the epoxy’s stiffness decreases significantly under elevated temperatures [9,32,47,48,49]. The literature reports that creep in concrete at an average temperature of 40 °C is about 25% higher than at 20 °C [50]. In another study, concrete subjected to 60 °C and sustained loading for 10 days exhibited approximately 50% higher total strain (elastic plus creep) compared to similar tests at 20 °C [49], with similar trends reported in other works [47,48]. The increase in CFRP strain is likely due to both the slab deformation and the higher thermal expansion of the epoxy adhesive, which is roughly four times that of concrete and much higher than that of CFRP. This mismatch in thermal expansion generates internal stresses between the CFRP and the adhesive, further contributing to the CFRP strain [51].

Comparing the TR and SS tests at 80 °C, several observations can be made regarding the monotonic tests:

- The SS slabs showed a slightly larger decrease in performance (≈9%) than the TR slabs, likely due to the longer exposure to elevated temperature.
- The TR slabs exhibited higher stiffness up to yielding, attributed to the preloading applied before the monotonic test.
- In the TR tests, the CFRP laminates reached lower strains than in the SS tests, due to residual deflection after unloading and the effects of sustained load and heating.
- Both the SS and TR slabs at 80 °C failed in the same cohesive mode.

Future experimental work could investigate the combined effects of sustained loading and elevated temperature on the NSM CFRP–concrete bond over longer periods, explore the temperature-dependent behaviour of different adhesives—including cement-based systems that may perform better at elevated temperatures—and evaluate the structural response of slabs under transient thermal conditions with varied preloading levels, as highlighted by the differences observed between the SS and TR tests.

## 4. Conclusions

This experimental study evaluated the flexural response of RC slab strips strengthened with NSM CFRP laminates when subjected to elevated service temperatures between 20 °C and 80 °C. Based on the experimental results, the following conclusions can be drawn:

1. Despite the epoxy adhesive having a *T_g_* of approximately 54 °C, the global structural response remained essentially unaffected up to 70 °C, with failure governed by concrete crushing. This indicates that the load transfer mechanism of the NSM system remained effective despite the progressive degradation of the adhesive.
2. At 80 °C, cohesive failure of the epoxy adhesive occurred, reducing the ultimate load capacity of the slabs by 11–13% in steady-state tests and by 1–3% in transient tests. These changes were associated with the degradation of the adhesive after exceeding the *T_g_*, highlighting the susceptibility of bond performance to temperature.
3. The SS slabs exhibited a greater reduction in maximum load (≈9%) and deflection at maximum load (≈12.5%) compared to the TR slabs at 80 °C. This was attributed to the longer exposure of the SS slabs to elevated temperature, highlighting the influence of thermal exposure duration on stiffness degradation and the flexural behaviour.

Overall, the results confirm that the NSM technique maintains satisfactory performance under elevated service temperatures up to 70 °C (~1.3 × *T_g_*), given the properties of the adhesive used in this study. However, adhesive degradation governs the structural response at higher temperatures and demands careful consideration in design and modelling. Furthermore, the pronounced increase in deflection observed at elevated temperatures highlights the potential governing of serviceability in the design of strengthening systems subjected to thermal action.

## Figures and Tables

**Table 1:** Experimental programme.

Type of Test	Slab ID	Sustained Load	Temperature Target [°C]
Steady-State (SS)	SL1_SS-20	-	20
	SL1_SS-40	-	40
	SL1_SS-50	-	50
	SL1_SS-70	-	70
	SL1_SS-80	-	80
	SL2_SS-80	-	80
Transient (TR)	SL1_TR-20	2/3 F_ult_	20
	SL1_TR-80	2/3 F_ult_	80 (heated in 4 h)
	SL2_TR-80	2/3 F_ult_	80 (heated in 12 h)

**Table 2:** The main results of the slabs obtained in the monotonic tests for both SS and TR slabs.

Slab ID	*δ*_cr_ [mm]	*F*_cr_ [kN]	*δ*_y_ [mm]	*F*_y_ [kN]	*δ*_max_ [mm]	*F*_max_ [kN]	ε_CFRP_ [‰]	ε_conc_ [‰]
SL1_SS-20	1.35	3.47	30.99	21.36	89.21	34.85	14.29	3.97
SL1_SS-40	1.67	3.64	30.47	20.97	83.92	35.74	13.71	3.27
SL1_SS-50	1.60	2.77	33.80	20.81	83.49	33.97	13.11	4.05
SL1_SS-70	2.08	2.78	31.61	19.84	81.16	31.73	12.11	3.69
SL1_SS-80	1.85	2.37	31.61	20.59	73.63	30.84	11.32 ^(b)^	2.97
SL2_SS-80	1.52	2.07	30.88	20.28	67.89	30.40	10.11 ^(b)^	2.99
SL1_TR-20	1.52 ^(a)^	3.72 ^(a)^	30.65 ^(c)^	23.93 ^(c)^	70.04	33.11	13.98 ^(b)^	4.12
SL1_TR-80	1.47 ^(a)^	4.43 ^(a)^	31.22 ^(c)^	24.84 ^(c)^	63.88	32.67	12.34	2.64
SL2_TR-80	1.62 ^(a)^	4.43 ^(a)^	33.47 ^(c)^	25.21 ^(c)^	61.97	32.03	12.71	2.36

Notes: *F*_cr_ = cracking load; *δ*_cr_ = mid-span deflection at *F*_cr_; *F*_y_ = steel yielding load; *δ*_y_ = mid-span deflection at *F*_y_; *F*_max_ = maximum load; *δ*_max_ = mid-span deflection at *F*_max_; *ε*_CFRP_ = CFRP strain at *F*_max_; *ε*_conc_ = mid-span concrete strain at *F*_max_; ^(a)^ values reached at the preloading phase; ^(b)^ strains measured at the point load section; ^(c)^ yielding of the reinforcement after the submission of a sustained loading.

## Data Availability

The original contributions presented in this study are included in the article. Further inquiries can be directed to the corresponding author.
